# The frequency of polycystic ovary syndrome in women with prediabetes compared with normoglycemic women

**DOI:** 10.3389/fendo.2025.1722978

**Published:** 2025-11-26

**Authors:** Cem Sulu, Ipek Pervaz, Turgut Gurer, Dogan Yildiz, Arzu Tas, Ahmet Numan Demir, Serdar Sahin, Hande Mefkure Ozkaya, Dildar Konukoglu, Abdullah Tuten, Taner Damci, Fahrettin Kelestimur, Mustafa Sait Gonen

**Affiliations:** 1 Division of Endocrinology-Metabolism and Diabetes - Department of Internal Medicine, Cerrahpasa Faculty of Medicine, Istanbul University-Cerrahpasa, Istanbul, Türkiye; 2 Cerrahpasa Faculty of Medicine, Istanbul University-Cerrahpasa, Istanbul, Türkiye; 3 Department of Internal Medicine, Cerrahpasa Faculty of Medicine, Istanbul University-Cerrahpasa, Istanbul, Türkiye; 4 Department of Biochemistry, Cerrahpasa Faculty of Medicine, Istanbul University-Cerrahpasa, Istanbul, Türkiye; 5 Department of Obstetrics and Gynecology, Cerrahpasa Faculty of Medicine, Istanbul University-Cerrahpasa, Istanbul, Türkiye; 6 Department of Internal Medicine, Division of Endocrinology and Metabolic Diseases, Faculty of Medicine, Yeditepe University, Istanbul, Türkiye

**Keywords:** hyperandrogenism, impaired fasting glucose, impaired glucose tolerance, insulin resistance, polycystic ovary syndrome, prediabetes

## Abstract

**Objective:**

To determine rate of polycystic ovary syndrome (PCOS) and its related features in women with prediabetes.

**Methods:**

Of 3465 consecutive women applied between 2021 and 2023, 3218 women with diabetes mellitus or conditions affecting gonadal functions were excluded through digital screening and tele-interviews. Remaining 247 women underwent clinical assessments, excluding another 49 due to other endocrine diseases. The diagnosis of PCOS and prediabetes were based on Rotterdam and American Diabetes Association criteria, respectively.

**Results:**

100 women had prediabetes and 98 women had normoglycemia. The frequency of PCOS were 17% and 19.4% in prediabetes and control groups, respectively (p=0.715). The frequency of PCOS was 24% (6/25) in women with impaired glucose tolerance (IGT) only, 22.2% (2/9) in women with impaired fasting glucose only, and 15.5% (9/58) in women who met the HbA1_C_ criterion only. Prediabetes group had higher insulin-like growth factor-1 (IGF–1) levels and lower anti-Müllerian hormone (AMH) levels (p<0.05). Insulin was correlated with testosterone, antral follicle count, and ovarian volume only in prediabetes group (p<0.05). Mediation models showed that insulin increased testosterone both directly and indirectly through increasing IGF-1 (b=0.4, p=0.0006).

**Conclusion:**

While the rate of PCOS was not increased in overall prediabetes group, a trend for an increased risk in IGT subgroup only was noteworthy. Positive correlation of insulin with testosterone, antral follicle count, and ovarian volume being only found in prediabetes group suggested that prediabetes might render ovaries susceptible to the PCOS-like changes. The lower AMH in prediabetes implied the toxic effects of even mild hyperglycemia on ovaries.

## 1 Introduction

Polycystic ovary syndrome (PCOS) is characterized by ovulatory dysfunction, clinical and/or biochemical hyperandrogenism, and polycystic ovarian morphology (1, 2). Its prevalence varies between 4% to 21%, depending on the criteria used for diagnosis and the population studied (3, 4). Although it is one of the most common endocrine disorders and the leading cause of hormone-related infertility in women, the etiology of PCOS is still elusive (4, 5). Overwhelming evidence suggest that insulin resistance (IR) and compensatory hyperinsulinemia play significant roles in the development of PCOS (6–8). Approximately 75% of women with PCOS have some level of IR which exposes them to a higher risk of a range of metabolic derangements including prediabetes, type 2 diabetes mellitus (T2DM), dyslipidemia, and cardiovascular complications (9–13).

Prediabetes is a highly prevalent condition commonly associated with IR and was coined to describe individuals with an intermediate state of glycemia in between normal glycemia and T2DM (14). The presence of PCOS in a woman with prediabetes increases the risk of developing overt diabetes by 5–10 times (12, 15). Addressing and treating PCOS in women with prediabetes may be instrumental for prompting expedited measures in the prevention of diabetes. This approach can also mitigate the negative effects of the PCOS on reproductive and general health in women of premenopausal age (16, 17).

A plethora of evidence revealed an increased prevalence of prediabetes in women with PCOS (11, 12, 18). On the other hand, the reverse scenario, namely the prevalence of PCOS in women with prediabetes, remains to be established. Since IR plays an important role in the pathogenesis of prediabetes and is also frequently associated with PCOS (6, 19), it is tempting to speculate that women with prediabetes would be at increased risk for PCOS. The primary objective of the present study is to shed light on the risk of PCOS in premenopausal women with prediabetes compared to women with normoglycemia. We also sought to assess the effect of the prediabetes on androgen levels, polycystic ovarian morphology, and ovulatory status.

## 2 Materials and methods

### 2.1 Study oversight

The present cross-sectional study was conducted in Endocrinology, Metabolism, and Diabetes outpatient clinics of tertiary care university hospital between July 2021 and December 2023. The study adhered to the STROBE guidelines.

### 2.2 Participants

Premenopausal women between 18–45 years of age who provided informed consent were included. Exclusion criteria were i) the presence of diabetes mellitus and other endocrine disorders (i.e., thyroid dysfunction, congenital adrenal hyperplasia, androgen secreting tumors, Cushing’s syndrome, hyperprolactinemia, and acromegaly) or systemic diseases that could affect hypothalamic-pituitary-gonadal axis, ii) inability to assess menstruation and/or ovulation status (i.e., prior hysterectomy, bilateral oophorectomy, and vaginal agenesis), iii) use of any medication that may interfere with carbohydrate metabolism and/or gonadal functions (i.e., oral antidiabetics, insulin, glucagon like peptide receptor agonists, glucocorticoids, oral contraceptive pills, antiandrogens, clomiphene citrate, gonadotropins, and letrozole) within the last 6 months preceding recruitment, iv) pregnancy, v) presence of intrauterine device, and vi) conditions that could interfere with HbA1c measurement.

### 2.3 Procedure

To ensure eligibility of each participant, a multi-step process was employed. The process began with digital review of 3465 consecutive seemingly healthy women who presented to the university hospital for a routine employment check-up between July 2021 and December 2023, during which 2384 women were excluded remotely using a standardized set of criteria mentioned above. Following the digital screening, we conducted telephone interviews on 1081 women and excluded 834 due to previously unregistered exclusionary criteria in the database. After the remote evaluations, the final stage involved in-person study assessments were performed on 247 pre-eligible women, detailed below.

i) Clinical and laboratory evaluations: Sociodemographic information was recorded, and detailed reproductive and family histories were obtained. Each woman underwent complete physical examination. Hirsutism was evaluated by an endocrinologist and quantified using the modified Ferriman-Gallwey (FG) scoring system (20). Female pattern hair loss and acne were assessed using the Global Acne Grading System (GAGS) and Ludwig Scale, respectively (21, 22).

Blood samples were obtained after 8 – h of fasting in early follicular phase (days 2 - 5) in women with regular menstrual cycles or oligomenorrhea and on a random day in those with amenorrhea. The plasma levels of total testosterone, free testosterone, androstenedione (A_4_), dehydroepiandrosterone sulfate (DHEA-S), 17-hydroxy progesterone (17-OH progesterone), sex hormone binding globulin (SHBG), follicle-stimulating hormone (FSH), luteinizing hormone (LH), estradiol, growth hormone (GH), insulin-like growth factor-1 (IGF-1), thyroid-stimulating hormone, free thyroxine, adrenocorticotropic hormone (ACTH), anti-Müllerian hormone (AMH), human chorionic gonadotropin, fasting blood glucose (FBG), glycosylated hemoglobin (HbA1c), and insulin were determined. Additional blood samples were collected in the mid - luteal phase from women with regular menstrual cycles to assess serum progesterone levels.

**Figure 1:**
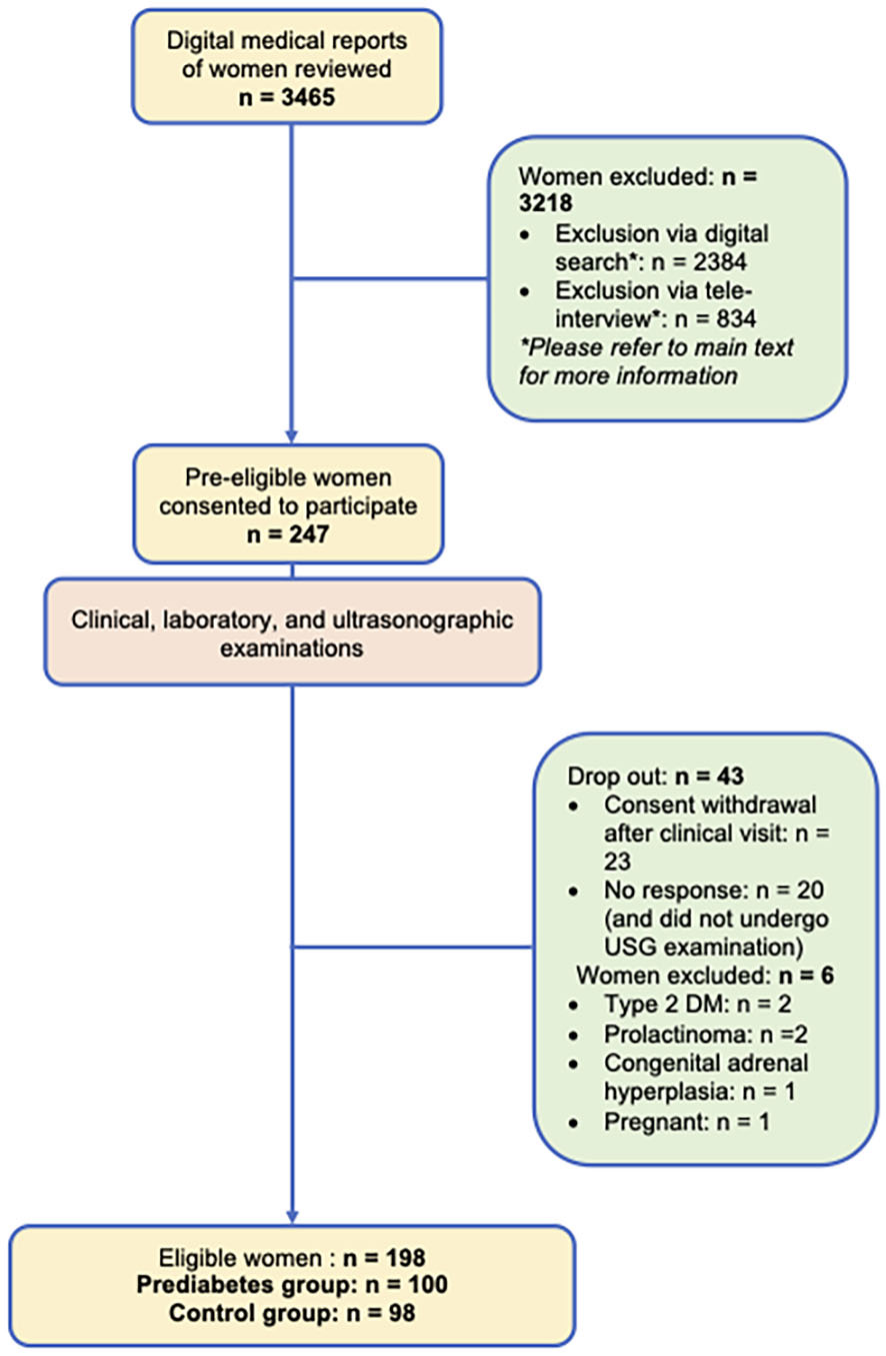
Flowchart of recruitment process.

Following the baseline blood sample collection, 75-g oral glucose tolerance test (OGTT) was performed and plasma glucose levels 120 minutes were recorded. High-dose (250 mcg) ACTH stimulation test was performed in four women with basal 17-OH progesterone > 2 ng/mL and one woman was excluded due to non-classical congenital adrenal hyperplasia (**Figure 1**). GH-OGTT suppression test was performed in one woman with basal IGF-1 levels higher than the upper limit of normal to exclude acromegaly. In 19 women with signs and symptoms suggestive of hypercortisolemia, 1 mg dexamethasone suppression test was performed Cushing’s syndrome was excluded.

The homeostasis model assessment (HOMA) of IR index was calculated based on the following formula: fasting insulin (mIU/L) x glucose (mg/dL)/405. The quantitative insulin sensitivity check index (QUICKI) was calculated according to the following equation: 1/[log insulin (mIU/L) + log glucose (mg/dL)].

ii) Ultrasonographic examination: Transvaginal ultrasound was performed by the same operator (AT^5^). The ovarian volumes and FNPO were recorded in each woman.

Finally, 198 women were classified into prediabetes and control groups. The recruitment process was illustrated in **Figure 1**.

### 2.4 Definitions

Impaired fasting glucose (IFG) was defined as fasting blood glucose 100–125 mg/dl; and impaired glucose tolerance (IGT) was 2-h glucose 140–199 mg/dl. Prediabetes was defined as the presence of IFG or IGT or HbA1c 5.7 – 6.4% (39 to 48 mmol/mol) (23).

The diagnosis of PCOS was based on revised Rotterdam criteria which requires the presence of minimum 2 of 3 of the following features: ovulatory dysfunction (OD), clinical hyperandrogenism and/or hyperandrogenemia (HA), and polycystic ovarian morphology (PCOM) (24). Ovulatory dysfunction was defined as the presence of oligomenorrhea (cycle length > 35 days) or amenorrhea (absence of menstruation for more than 6 months) or luteal progesterone level < 5 ng/mL (25).

Diagnosis of clinical hyperandrogenism was based on modified FG score ≥ 8 (26). Biochemical hyperandrogenemia was defined as the elevated levels of serum total testosterone (normal range: 8.4 – 48.1 ng/dL) or free testosterone (normal range: 0,01 – 4,2 pg/mL) or DHEA-S (normal range: 60.9 – 337 µg/dL) or androstenedione (normal range: 1,71 – 4,58 ng/mL) beyond their respective normal ranges. Follicle number per ovary (FNPO) ≥ 20 in at least one ovary and/or ovarian volume (OV) ≥ 10ml was considered threshold for polycystic ovarian morphology (PCOM) (24).

Overweight was defined as BMI between 25 and 29.9 and obesity as BMI ≥ 30.

### 2.5 Assays

The serum levels of hormones were performed using electrochemiluminescence immunoassay (ECLIA) method. Roche/Cobas e system (Cobas e 602, Roche Diagnostics GmbH, Mannheim, Germany) was used to determine total testosterone, androstenedione, DHEA – S, AMH, progesterone, estradiol, FSH, LH, GH, and IGF – 1 levels. Snibe Diagnostics immunoassay system (MAGLUMI 2000, Snibe Diagnostics, China) was used to determine serum levels of 17 – OH progesterone and serum free testosterone. Plasma glucose and lipid levels were determined by Roche/Cobas c systems according to the manufacturer’s kits and recommendations using enzymatic colorimetric methods (Roche/Cobas c 702, Roche Diagnostics GmbH, Mannheim, Germany). HbA1c levels were analyzed using ion exchange chromatography method (Lifotronic Technology Co., Ltd. H9 HPLC analyzer China).

### 2.6 Statistical analyses

Statistical analyzes were performed using SPSS version 22.0. Data were presented as numbers and percentages, means and standard deviations, or median and interquartile range [IQR]. Normal distribution was analyzed using the Kolmogorov–Smirnov test. Univariate analysis was carried out using the Pearson chi-square test for the categorical variables, independent samples t test and Mann–Whitney U test for the normally distributed continuous variables, and the Mann–Whitney U test for the non-normally distributed continuous variables. SPSS PROCESS macro was used to test hypotheses on the moderation and mediation effects. Briefly, a mediation model is used to investigate the process through which an independent variable influences a dependent variable by including one or more intermediate variables, known as mediators. The intent is to understand the mechanism or pathway by which the independent variable affects the dependent variable. Moderation model is used to investigate whether the strength or direction of the relationship between independent and dependent variables is influenced by the level of a third variable, known as the moderator. The moderator variable helps identify the conditions under which the relationship between the main variables become more or less evident. P-values < 0.05 were considered significant.

Since no previous study has reported the prevalence of PCOS among women with prediabetes, sample size estimation was derived from data on women with type 2 diabetes mellitus, in whom PCOS prevalence is approximately 20%, compared with 7% in the general population. Using the approximately 13% difference in PCOS prevalence reported between women with type 2 diabetes (20%) and the general population (7%) as a reference, and assuming α = 0.05 and 80% power, the required minimum sample size was estimated to be 90 participants per group.

## 3 Results

### 3.1 Clinical and laboratory features of the study groups

One hundred women with prediabetes and 98 control subjects were included in the final analyses. In the prediabetes group, 25 women had IGT only (25%), 9 women had IFG only (9%), 58 women (58%) fulfilled HbA1c criterion only, 8 women had both IGT and IFG (8%).

Sociodemographic and clinical features of study groups are presented in **Table 1**. The women with prediabetes were older than the women in the control group (36 ± 7 vs 33 ± 7 years, p = 0.009). Body mass index (BMI) was higher in the prediabetes group compared to the control group (28.8 ± 6 vs 25.9 ± 4.3, p < 0.001).

**Table 1:** Demographics and clinical features of study groups.

Feature	Prediabetes* group n=100			Control group n=98			P - value
Age, years	36	±	7	33	±	7	0.009
Marital status, n (%)							0.461
*Married*	70		(70)	62		(63.3)	
*Single*	28		(28)	35		(35.7)	
*Divorced*	2		(2)	1		(1)	
Menarche, years	13.1	±	1.5	13.2	±	1.5	0.763
Gravidity	2		[0 – 3]	1.5		[0 – 2]	0.179
Parity	2		[0 – 2]	1		[0 – 2]	0.051
Prior birth control technique, n (%)							0.249
*None*	37		(37)	49		(50)	
*Barrier methods*	12		(12)	12		(12.2)	
*Oral contraceptive*	14		(14)	5		(5.1)	
*Intrauterine device*	10		(10)	12		(12.2)	
*Coitus interruptus*	9		(9)	10		(10.2)	
*Ovarian tubal ligation*	7		(7)	2		(2)	
*Oral contraceptive - barrier methods*	2		(2)	0		(0)	
*Oral contraceptive - intrauterine device*	6		(6)	4		(4.1)	
*Oral contraceptive – coitus interruptus*	1		(1)	1		(1)	
*Intrauterine device - tubal ligation*	2		(2)	2		(2)	
*Intrauterine device - coitus interruptus*	0		(0)	1		(1)	
Prior assisted reproductive technique, n (%)	6		(6)	5		(5.1)	1.0
Family history, n (%)							
*Hirsutism*	6		(6)	10		(10.2)	0.308
*Acne*	6		(6)	12		(12.2)	0.144
*Alopecia*	6		(6)	11		(11.2)	0.214
*Infertility*	13		(13)	8		(8.2)	0.357
*Assisted reproductive technique*	6		(6)	6		(6.1)	1.0

*American Diabetes Association criteria.

Continuous data were presented as mean ± standard deviation or median [interquartile range] depending on distribution.

HOMA and QUICKI scores showed marginally non-significant differences (p = 0.054 for both), indicating a trend for higher IR in the prediabetes group compared to the control group (**Table 2**). The prediabetes group had higher FSH and IGF-1 levels than the control group (p = 0.001 and p = 0.013, respectively). SHBG and AMH levels were lower in the prediabetes group than the control group (p = 0.003 and p = 0.016, respectively). The groups were similar with respect to other laboratory parameters (**Table 2**).

**Table 2:** Laboratory parameters of the study groups.

Feature	Prediabetes group			Control group			P - value
Fasting blood glucose, mg/dL	83.8	±	17.4	75.6	±	10.4	0.001
2h glucose, mg/dL	115		[93 – 150]	91		[79 – 104]	0.001
HbA_1C_ %	5.9		[5.7 – 6]	5.4		[5.3 – 5.6]	0.001
Insulin, pmol/L	11.6		[6.4 – 18.1]	10.9		[7.8 – 13.2]	0.308
HOMA-IR	2.5		[1.3 – 4]	2		[1.2 – 2.8]	0.054
QUICKI	0.33		[0.31 – 0.37]	0.34		[0.33 – 0.37]	0.054
Total cholesterol, mg/dL	179	±	34.5	172	±	33.4	0.321
LDL-cholesterol, mg/dL	113	±	29	107	±	34	0.385
HDL-cholesterol, mg/dL	52		[45 – 58]	56		[47 – 66]	0.034
Triglycerides, mg/dL	94		[70 – 138]	83		[61 – 99]	0.007
Total testosterone, ng/dL	26.1	±	15	29.8	±	15	0.569
Free testosterone, pg/mL	1.8		[1.5 – 2.3]	1.7		[1.5 – 2.2]	0.584
DHEA-S, mcg/dL	200		[131 – 273]	206		[147 – 138]	0.631
Androstenedione, ng/mL	1		[0.7 – 1.4]	0.8		[0.8 – 1.5]	0.302
SHBG, mg/L	3.7		[2.4 – 5.2]	4.7		[3.2 – 6.4]	0.003
17-OH progesterone, ng/mL	0.7		[0.5 – 1.0]	0.8		[0.6 – 1.0]	0.221
Progesterone, ng/mL	4.7		[0.4 – 11.6]	5.7		[0.4 – 10.6]	0.936
AMH, pmol/L	6.9		[2.9 – 15.8]	10.8		[5 – 22.5]	0.016
ACTH, pg/mL	18.8		[13.3 – 29.4]	21.9		[14.2 – 38.7]	0.274
Cortisol, mcg/dL	12.9		[9.2 – 16.2]	11.3		[9.3 – 15.7]	0.406
Prolactin, ng/mL	16		[11 – 23]	16		[12 – 21]	0.338
FSH, mIU/mL	6.8		[5.5 – 8.7]	5.7		[4.7 – 7.3]	0.001
LH, mIU/mL	5.8		[4.4 – 7.7]	6		[3.8 – 8.7]	0.824
Estradiol, pg/mL	45		[33 – 64]	51		[35 – 82]	0.093
TSH, mIU/L	2.1		[1.6 – 3.3]	2.6		[1.8 – 3.2]	0.395
Free T4, ng/dL	1.2	±	0.14	1.2	±	0.15	0.670
Growth hormone, ng/mL	0.5		[0.2 – 1.7]	0.8		[0.3 – 2.7]	0.064
IGF-1, ng/mL	129		[109 – 187]	117		[117 – 198]	0.013

Data were presented as mean ± standard deviation or median [interquartile range] depending distribution

*American Diabetes Association criteria

ACTH, Adrenocorticotropic hormone, AMH, Anti-Müllerian hormone, DHEA-S, Dehydroepiandrosterone-sulfate, FSH, Follicle-stimulating hormone, HDL, High-density lipoprotein, HOMA – IR, homeostatic model assessment - Insulin resistance, LDL, Low-density lipoprotein, LH, Luteinizing hormone, IGF-1, Insulin-like growth factor-1, TSH, Thyroid-stimulating hormone; QUICKI, quantitative insulin sensitivity check index

The frequency of PCOS was 17% (n = 17) in the prediabetes group and 19.4% (n = 19) in the control group (p = 0.715). The frequency of PCOS was 24% (6/25) in women with IGT only, 22.2% (2/9) in women with IFG only, and 15.5% (9/58) in women who met the HbA1_C_ criterion only. The pairwise comparison within prediabetes subgroups showed no significant differences in PCOS prevalence (p > 0.05 for all).

The prediabetes and control groups were not different in frequencies of hyperandrogenemia, hirsutism, androgenic female pattern hair loss, acne, and PCOM (**Table 3**).

**Table 3:** Comparison of PCOS prevalence and PCOS-related features.

All participants	Prediabetes group (n = 100)	Control group (n = 98)	P-value
PCOS, n (%)	17 (17)	19 (19.4)	0.715
*Phenotype A*	4 (4)	5 (5.1)	0.746
*Phenotype B*	8 (8)	5 (5.1)	0.410
*Phenotype C*	3 (3)	3 (3.1)	1.0
*Phenotype D*	2 (2)	6 (6.1)	0.168
Hirsutism*, n (%)	17 (17)	10 (10.2)	0.214
Ludwig Scale, n (%)			0.950
*Stage I*	14 (14)	14 (14.3)	
*Stage II*	2 (2)	2 (2)	
*Stage III*	0	0	
Global Acne Grading System			0.897
*Mild*	17 (17)	15 (15.3)	
*Moderate*	0	1 (1)	
*Severe*	0	1 (1)	
Biochemical hyperandrogenism, n (%)	17 (17)	16 (16.3)	1.0
Polycystic ovarian morphology, n (%)	21 (21)	32 (32.7)	0.078
Ovulatory dysfunction, n (%)	26 (26)	25 (25.5)	1.0

*Modified Ferriman-Gallwey score ≥ 8.

PCOS, Polycystic ovary syndrome.

#### 3.1.1 The effect of age on PCOS frequency

To address the concern regarding potential confounding by age, we performed an additional analysis using case–control matching based on age (1:1 nearest-neighbor matching without replacement, tolerance ±2 years). After matching, both groups were perfectly balanced in terms of age (mean age 34.97 ± 7.35 vs. 34.94 ± 7.58 years, p = 0.97). In this age-matched subset (n = 158), the prevalence of polycystic ovary syndrome remained similar between women with prediabetes and normoglycemic controls (15.2% vs. 19.0%, p = 0.53).

### 3.2 Relations of markers of IR with PCOS-related parameters

The relationship between markers of IR (serum insulin level, HOMA and QUICKI scores) and PCOS-related parameters (testosterone level, OV, FNPO) were analyzed. The markers of IR were positively correlated with testosterone levels, FNPO, and OV in the prediabetes group but not in the control group (**Table 4**).

**Table 4:** Correlations of markers of IR with androgen levels, FNPO, and ovarian volume.

Markers of IR	Total testosterone		Free testosterone		FNPO		Ovarian volume	
Prediabetes group	r	*p*	r	*p*	r	*p*	r	*p*
Insulin	0.558	0.009	0.671	0.001	0.360	<0.001	0.240	0.02
HOMA	0.523	0.015	0.623	0.003	0.305	0.003	0.237	0.022
QUICKI	-0.498	0.022	-0.538	0.012	-0.307	0.003	-0.239	0.021
Control group								
Insulin	0.025	0.806	-0.004	0.967	0.029	0.782	0.047	0.659
HOMA	0.023	0.825	-0.037	0.717	0.047	0.652	0.055	0.609
QUICKI	-0.023	0.825	0.037	0.717	-0.047	0.652	-0.055	0.609

r: Spearman’s rank correlation coefficient.

FNPO, Follicle number per ovary; FSH, Follicle stimulating hormone; HOMA, Homeostatic model assessment; LH, Luteinizing hormone; QUICKI, Quantitative insulin sensitivity check index, IR, Insulin resistance.

Since the correlations between markers of IR and androgen levels were present only in the prediabetes group, we speculated that the higher BMI in the prediabetes group might have influenced the relationship between IR and androgen levels. The moderation analysis showed that BMI neither had significant effect on serum testosterone levels (b = 0.03, t (44) = 1.6, p = 0.1, 95% CI [-0.007 – 0.06]) nor influenced relationship between insulin and testosterone (b = -0.02, t (44) = -1.6, p = 0.12, 95% CI [-0.004 – 0.0004]). On the other hand, we found that insulin is a significant predictor of serum testosterone (b = 0.07, t (44) = 2.1, p = 0.04, 95% CI [0.003 – 0.13]), and higher insulin levels are associated with higher levels of testosterone in the prediabetes group. When controlling for age, insulin remained a significant predictor of free testosterone (B = 0.014 ± 0.006, p = 0.034). These results suggested that the effect of insulin on testosterone levels was independent of BMI in the prediabetes group.

**Figure 2:**
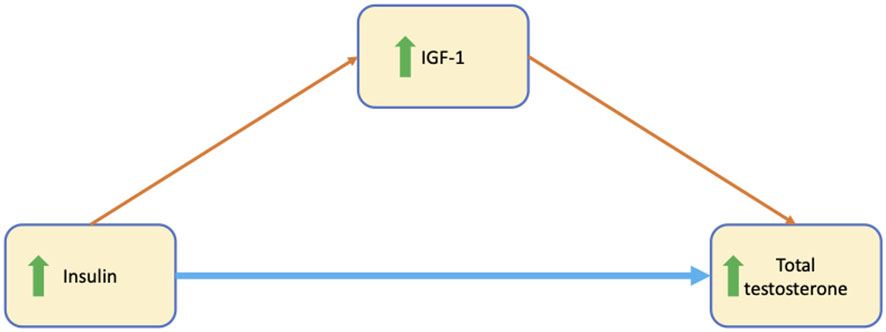
Mediation model depicting direct and indirect effects of insulin on serum testosterone levels. The blue line designates the significant direct effect of insulin on testosterone, indicates that increased insulin directly increase testosterone levels. The orange lines denotes the effect of insulin on IGF-1, showing increased insulin was related to increased IGF-1. IGF-1 was a strong predictor of total testosterone and served as mediators of the relationship between insulin and total testosterone levels.

IGF-1 levels were positively correlated with both insulin and testosterone levels in women with prediabetes (r = 0.265, p = 0.008; r = 0.435, p < 0.001, respectively) but not in those without prediabetes. Therefore, we analyzed whether IGF-1 had mediating roles in the relationship between insulin and androgen levels in women with prediabetes. The mediation model indicated that elevated insulin increases serum total testosterone directly, and indirectly through increasing IGF-1 in women with prediabetes (**Table 5**, **Figure 2**). In this reverse model (testosterone → IGF-1 → insulin), testosterone was associated with IGF-1 (β = 0.28, p = 0.005), but IGF-1 was not associated with insulin (p = 0.122). The indirect effect of testosterone on insulin through IGF-1 was not statistically significant (95% CI: –0.28 to 1.75).

**Table 5:** Results of the mediation analyses for direct and indirect effects of insulin on total testosterone in prediabetes group.

Effect of insulin on mediators	Coefficient	%95 CI	*R^2^*	p
* Insulin → IGF-1*	1.1	0.2 – 2	6%	0.019
Effect of mediators on testosterone	Coefficient	%95 CI		p
*IGF-1 → Total testosterone*	0.09	0.04 – 0.13		0.0005
Effect of insulin on total testosterone	Coefficient	%95 CI		p
*Total effect*	0.40	0.2 – 0.6		0.0006
*Direct effect*	0.30	0.1 – 0.5		0.006
Indirect effect via*:				
*IGF-1*	0.10	0.005 – 0.3		

*R^2,^* Proportion of mediation.

*Significance of indirect effect is determined when CIs do not encompass “0”.

CI, Confidence interval; IGF-1, Insulin-like growth factor-1.

## 4 Discussion

This study investigated the frequency of PCOS in women with prediabetes. We found a PCOS prevalence of 17% in women with prediabetes, which did not differ compared to women without prediabetes. Insulin resistance was slightly higher in women with prediabetes and positively correlated with testosterone levels, follicle number per ovary, and ovarian volume exclusively in the prediabetes group. These results suggested that while presence of prediabetes per se is not sufficient for an increase in the risk of PCOS, it may render ovaries susceptible to the hormonal and morphological changes associated with PCOS.

Prior studies reported a 2–5-fold higher risk of glucose metabolism disorders in PCOS (11, 12, 18), suggesting a shared pathophysiologic background in insulin action and secretion (7, 27, 28). Consistent with this hypothesis, T2DM has been reported to be associated with increased risk for PCOS and PCOM (29, 30). Since prediabetes is also commonly accompanied with IR, a higher prevalence of PCOS in women with prediabetes would also be anticipated. Nevertheless, the results of this study did not reveal an increased risk of PCOS in the prediabetes group. As IR is a continuum rather than a binary parameter, its severity correlates with the degree of clinical expression of PCOS (31, 32). In our study, the IR were only mildly elevated in the prediabetes group, and serum insulin levels were similar between the groups. This relatively modest increase of IR in the prediabetes group might have fallen short to promote the full-blown clinical expression of PCOS.

According to ADA criteria, prediabetes comprises heterogeneous subtypes, rendering it an umbrella term covering various phenotypes with distinct pathophysiologic backgrounds that reflect different aspects of glucose metabolism (33–36). In this study, more than half of the women in the prediabetes group fulfilled the HbA1c criterion only. In other words, vast majority of women in the prediabetes group probably had subtle defects in insulin action and/or secretion but still had hyperglycemia. The women with IGT only comprised just a quarter of prediabetes group in our study, and 24% of them had PCOS. This prevalence was higher than 15.5% in women with HbA1c criterion only, supporting the notion that IGT represents a more advanced stage in prediabetes and therefore might confer a higher risk of PCOS.

In this study, there was a trend for higher HOMA-IR and lower QUICKI scores in the prediabetes group compared to control group, suggesting a mildly increased IR in women with prediabetes. These parameters were positively correlated with serum testosterone, antral follicle count, and ovarian volume in women with prediabetes. On the other hand, IR showed no correlation with serum androgen levels or antral follicle count in those without prediabetes. One explanation for this finding could be that IR triggers ovarian hormonal and morphological changes above a particular threshold. The lower degree of IR in the control group compared to the prediabetes group might have fallen below that putative threshold. Therefore, we speculated that while the IR in women with prediabetes might not end up with a clear-cut PCOS phenotype, it might still promote PCOS-like ovarian changes once it reaches a certain point.

An alternative explanation for the correlation of IR with total testosterone levels being found exclusively in the prediabetes group might be related to the ovarian hypersensitivity to insulin under chronic hyperglycemia. Oxidative stress induced by hyperglycemia may amplify insulin’s steroidogenic effect and promote theca-cell proliferation (37, 38). Moreover, our finding of low AMH levels in the prediabetes group, suggesting a diminished ovarian reserve in women with prediabetes, might lend further support to the potential toxic effects of hyperglycemia on the ovaries.

The findings of the present study align with accumulating evidence that hyperinsulinemia, IGF-1 signaling, and androgen excess constitute an interconnected triad in PCOS pathophysiology (39). Insulin and IGF-1 share overlapping receptor-mediated pathways that converge on theca cell steroidogenesis (40). Hyperinsulinemia suppresses hepatic insulin-like growth factor binding protein-1 synthesis, increasing bioavailable IGF-1, which in turn acts synergistically with LH to enhance ovarian androgen production (41). A recent meta-analysis indicated that IGF-1 levels are elevated in women with PCOS, particularly in non-obese phenotypes, supporting its role as a metabolic amplifier of androgen synthesis (42). In our cohort, IGF-1 levels were higher in women with prediabetes and mediated the effect of insulin on testosterone, suggesting that early dysglycemia may already activate this insulin–IGF-1–androgen axis. It is plausible that lower IGF-1 levels in normoglycemic women may have attenuated the effect of insulin on testosterone, thereby abolishing this correlation in the control group. Although IGF-1 may not represent a primary etiological driver (43, 44), its contribution to androgen biosynthesis underscores the importance of targeting insulin and IGF-1 pathways to prevent the reproductive and metabolic complications of prediabetes.

From a therapeutic perspective, our findings suggest that targeting IR in women with prediabetes could provide both metabolic and reproductive benefits (45). Insulin-sensitizing therapies such as metformin may exert a threefold effect by lowering the risk of diabetes progression, reducing insulin-mediated ovarian androgen synthesis, and decreasing IGF-1 levels (46, 47). Similarly, inositols and central opioid receptor modulators have shown promise in improving insulin sensitivity and restoring ovulatory function (48). These insights underscore the importance of early metabolic interventions not only for diabetes prevention but also for mitigating the subclinical androgenic consequences of prediabetes.

This study has several limitations. First, its cross-sectional design precludes causal inferences regarding the metabolic, hormonal, and structural effects of prediabetes on the ovaries. Second, the relatively modest sample size, particularly within IFG and IGT subgroups, may have limited the power to detect small but clinically meaningful differences. Third, despite careful exclusion criteria, some degree of residual confounding related to age, BMI, or lifestyle factors cannot be fully ruled out. Fourth, the single-center design may limit the generalizability of our findings to other populations or ethnic groups. Finally, although all laboratory analyses were performed using standardized assays, dynamic tests of insulin sensitivity and secretion were not included, which might have provided a more comprehensive assessment of insulin physiology in women with prediabetes.

## 5 Conclusions

The current study provided information on the risk of PCOS in women with prediabetes. While prediabetes seemed not to be a primary risk factor for PCOS, it could potentially expose ovaries to the hormonal and morphological alterations suggestive of PCOS. In women with prediabetes, a slightly higher IR or an increased sensitivity of ovaries to the effects of insulin might be responsible for the dynamics between insulin and ovaries. Toxic effects of hyperglycemia on ovaries and/or increased IGF-1 levels might potentiate the effects of insulin on ovaries in women with prediabetes. The understanding of the complex relationship between PCOS and prediabetes requires more extensive research.

## Data Availability

All data generated or analyzed during this study are included in this article and its tables. Further inquiries can be directed to the corresponding author. M.S.G. is the guarantor of this work and, as such, had full access to all the data in the study and takes responsibility for the integrity of the data and the accuracy of the data analysis.
